# Measuring the Manipulation of T Helper Immune Responses by *Schistosoma mansoni*

**DOI:** 10.3390/ijms23031462

**Published:** 2022-01-27

**Authors:** Mebrahtu G. Tedla, Alison L. Every, Jean-Pierre Y. Scheerlinck

**Affiliations:** 1 Centre for Animal Biotechnology, Faculty of Veterinary and Agricultural Sciences, The University of Melbourne, Parkville, VIC 3010, Australia; A.Every@latrobe.edu.au (A.L.E.); j.scheerlinck@unimelb.edu.au (J.-P.Y.S.); 2 Office of the Provost, La Trobe University, Bundoora, VIC 3086, Australia

**Keywords:** immune memory, in vitro, in vivo, OVA, resilience, *S. mansoni*, Th memory cells

## Abstract

*Schistosoma mansoni* uses different mechanisms to escape its host’s immunity. Understanding the ability of memory T cells to withstand this pathogen’s manipulation is important for the development of effective vaccines against this immunomodulatory pathogen. In this study, ovalbumin (OVA) transgenic *S. mansoni* is used as a tool to investigate whether fully differentiated Th1, Th2 and Th17 cells are able to withstand pathogen manipulation. Naïve T cells from OT-II T cell receptor transgenic mice with a specificity for OVA were differentiated into Th1, Th2, and Th17 polarised memory cells in vitro. These cells were adoptively transferred into recipient mice to investigate whether these polarised immune memory T cells are resilient in the face of pathogen-mediated manipulation. After transferring memory cells, mice were challenged with OVA-transduced *S. mansoni* eggs as well as wild-type controls. The in vitro differentiated Th1, Th2 and Th17 memory cells continued to produce the same cytokines when challenged by OVA-expressing *S. mansoni* eggs as to these they produced when transferred in vivo, suggesting that the Th phenotypes of the memory T cells remains unaltered in the face of stimulation by *S. mansoni*. The ability of memory T cells to remain resilient to manipulation by the parasite suggests that vaccines might be able to produce immune memory responses able to withstand *S. mansoni* immune manipulation and hence protect the host from infection.

## 1. Introduction

Long-lasting protective immunity against a pathogen, in a host, is most often achieved through the development of pathogen-specific immune memory T cells that can be restimulated during the early phase of the infection. The functional properties of such memory T cells are orchestrated by cellular and molecular regulators, which define their phenotype and might also regulate their plasticity (i.e., the extent to which their phenotype can still be influenced by the pathogen) [1]. The functional characteristics of memory T cells such as their plasticity, their ability to be recalled, and the duration of their persistence have significant implications for vaccination strategies. Indeed, for vaccines to be effective the immune memory needs to be resilent to manipulation by the pathogen during infection, so that the host is protected from pathogen-mediated manipulation of the burgeoning immune response, and persistence and recall functions of the immune response is not compromised during infection [1].

The complex biology of parasites has often, over evolutionary timeframes, allowed them to develop specific mechanisms to manipulate the host’s immune system in order survive long-term. For example, *S. mansoni* secretes immunomodulatory molecules able to impact DC functions [2] and affect the type of T helper responses by inducing nonspecific suppressive pathways during different life stages [3]. Indeed, *S. mansoni* secretory excretory antigens (SEA), produced in the egg-stage of the parasite, have the ability to manipulate human or mouse DCs to suppress classical maturation of co-stimulatory ligand expression. These manipulated DCs induce a Th2 response in vitro following a transfer to a naïve host under MyD88^-^ and IL-4-independent conditions [4]. Molecules having an effect on this pathway have been identified, including *S. mansoni* Omega-1, resulting in the induction of a Th2 type immune response [5,6]. While the mechanisms of T cell manipulation by *S. mansoni* eggs are well understood for naïve T cells, it is unclear whether this manipulation takes place against fully differentiated immune memory T cells with an established T helper phenotype bias. In turn, this understanding of whether parasites are able to influence an established immune memory response during the recall phase is critical to vaccination strategies relying on immune memory T cells able to withstand pathogen manipulation.

In this study, we utilise a model based on OT-II cells and transgenic *S. mansoni* parasites to investigate whether fully differentiated Th1, Th2 and Th17 immune memory cells can withstand immune manipulation by *S. mansoni* parasites following in vivo challenge. This work may also have important implications for the use of parasite-derived molecules as therapeutic agents [7,8,9,10].

## 2. Materials and Methods

### 2.1. Experimental Mice

Six- to eight-week-old female BALB/c and OT-II mice were purchased from the Walter and Eliza Hall Institute of Medical Research (WEHI, Melbourne, Australia) and were used in the present study to maintain the life cycle of *S. mansoni* and as a source of naïve OT-II T cells, respectively. All the experiments related to mice were conducted subject to approval from the Animal Ethical Committee of the University of Melbourne (Ethics ID: 1312952).

### 2.2. Transduction of S. mansoni Eggs Using Lentivirus Vector

We followed the egg-transduction method of Hagen et al. [11]. Depending on the purpose, a range of 8000 to 12,000 eggs were used for viral transfection and untranduced (wild type) eggs were also included as a control. The eggs were cultured with 500 μL complete DMEM in the presence of 100 μL virus particles at a titter of 25 × 10^3^ pfu per ml of OVA-encoding pGIPZ lentiviral vector containing CMV promoter or left untreated in complete DMEM. The OVA expressing construct was designed so that parasite-expressed OVA has an identical protein sequence as native OVA [12]. The OVA transduced parasite eggs where previously characterized extensively and were shown to stimulate OT-II cells [12].

### 2.3. Cell Culture

Spleen, maxillary and inguinal lymph nodes of BALB/c and OT-II mice were collected and pooled in RPMI-1640 (Invitrogen, Life Technologies, Waltham, MA, USA). The tissues were crushed using forceps and filtered through a 70 µm nylon cell strainer. The cells were centrifuged at 276× *g* for 6 min. After aspirating the supernatants, the red blood cell pellets were lysed using distilled water for 9 s, followed by addition of 10% of 10× PBS to stop the lysis reaction. The cell suspension was centrifuged at 276× *g* for 6 min. Supernatants were removed, and the cells were resuspended in fresh complete RPMI-1640 medium ((Invitrogen, Life Technologies) supplemented with 2 mM L-glutamine, 100 U/mL penicillin, 100 μg/mL streptomycin and 10% *v*/*v* heat-inactivated FCS, and 50 μM 2-mercaptoethanol). When required, cell concentrations were estimated using a hemocytometer. In all experiments we used pools of cells prepared from spleen and lymph nodes.

### 2.4. In Vitro Polarization of OT-II T Cells

Naïve OT-II cells prepared as above, were polarized into three lineages using differentiating cytokines as follows: 2 × 10^5^ cells per well were cultured for 4 days and polarized into Th1 (using 10 μg/mL OVA peptide, 10 ng/mL of IL-2), Th2 (using 10 μg OVA peptide and 10 ng/mL of IL-2, 10 ng/mL of IL-4 and 10 μg/mL of anti-IFN-γ), and Th17 (using 10 μg/mL OVA peptide, 10 μg/mL of anti-IL-4, 10 μg/mL of anti-IFN-γ antibodies, 5 ng/mL of anti-TGFβ and 20 ng/mL of IL-6). Naïve cells were stimulated with 10 μg/mL of OVA peptide only, to get unpolarized cells. Cells were further stimulated for two days using 4.5 µg/mL of IL-7 (PeproTech, Rocky Hill, NJ, USA) to generate memory cells. Th-polarised cells, 10^5^, per well were stimulated with OVA peptide (10 μg/mL) for 72 h. The degree of polarization was determined based on the cytokine signatures of the differentiated cells using the mouse Th1, Th2, and Th17 cytometric bead array (BD Bioscience, North Brunswick Township, NJ, USA) as per manufacturer’s instructions.

### 2.5. Adoptive Transfer of OT-II T Cells to Congenic Mice and Parasite Injection

A total of 2 × 10^6^ carboxyfluorescein-diacetate-succinimidyl-ester (CFSE; Bio Legend, San Diego, CA, USA) labelled OT-II T cells, which express CD45.2, were adoptively transferred into CD45.1 congenic mice. After 48 h, mice were injected with different antigens depending on the purpose of the experiment, including 1000 transduced or untransduced *S. mansoni* eggs suspended in 50 µL of PBS through intravenously (IV), or 20 µg/mL of OVA peptide as a positive control through the same route. Similarly, unchallenged OT-II T cells were used as a negative control. After 7 days, mice were culled to extract OT-II T cells from spleen for further analysis.

### 2.6. T Cell Proliferation Assay

Spleen, maxillary, axillary and inguinal lymph nodes of OT-II mice were collected and pooled for cell extraction using complete RPMI-1640 medium (Invitrogen, Life Technologies). Single cell suspensions were obtained by passing the tissue through a 70 µm nylon cell strainer. Red blood cells were lysed by water and lysis was stopped using 10× PBS, as described above. Cells were then resuspended in complete RPMI-1640 medium. For proliferation assays, cells were stained with CFSE according to manufacturer’s instruction. A total of 2 × 10^5^ labelled cells per well were co-incubated with 25, 50, 100, or 200 transduced or un-transduced *S. mansoni* eggs for 4–5 days in 96 well plates at 37 °C, 5% CO_2_. After four days culturing, OT-II cells were collected and washed with FACS buffer (10% FCS in PBS). For proliferation analysis, cells were stained with PE-anti-mouse CD4 (Bio Legend) following an FcγR-surface block (anti-FcγR, clone 2.4G2-16, WEHI Monoclonal Facility). To exclude dead cells, 7-amino-actinomycin D (1 µg/mL; Sigma Aldrich, Steinheim, Germany) was added to cell suspension prior data acquisition using The BD FACSVerse™ flow cytometer (BD Bioscience). The data was analysed using flowjo version 10 (BD Bioscience, Ashland, OR, USA) gating on CD4^+^ T cells having CFSE^low^ phenotypes. In the standard technique for cell proliferation the presence of low levels of CFSE indicate that the cells have proliferated since the dye has been diluted during consecutive cell divisions. The results were expressed as % CFSE^low^ cells in the total CD4 population.

### 2.7. Cytokine Assay

Cell culture supernatants from T cell proliferation assays were analysed for cytokine production by mouse Th1, Th2, and Th17 cytometric bead array (BD Bioscience) as per the manufacturer’s instructions. According to the manufacturer, the detection limits for each cytokine were: IL-2 (0.1 pg/mL), IL-4 (0.03 pg/mL), IL-6 (1.4 pg/mL), IFN-γ (0.5 pg/mL), TNF (0.9 pg/mL), IL-17 A (0.8 pg/mL) and IL-10 (16.8 pg/mL).

### 2.8. Statistical Analysis

Data was computed and analysed using the single cell analysis software: flowjo version 10. All statistical analyses were performed using one-way ANOVA followed by *t*-test using Graph Pad Prism version 7.00 for Windows (GraphPad Software, La Jolla, CA, USA). In all the analyses, the confidence level was held at 95% and *p* < 0.05 was required for significance.

## 3. Result

### 3.1. In Vitro Polarization of Naïve OT-II T Cells and Analysis of Their Cytokine Signatures

**Figure 1:**
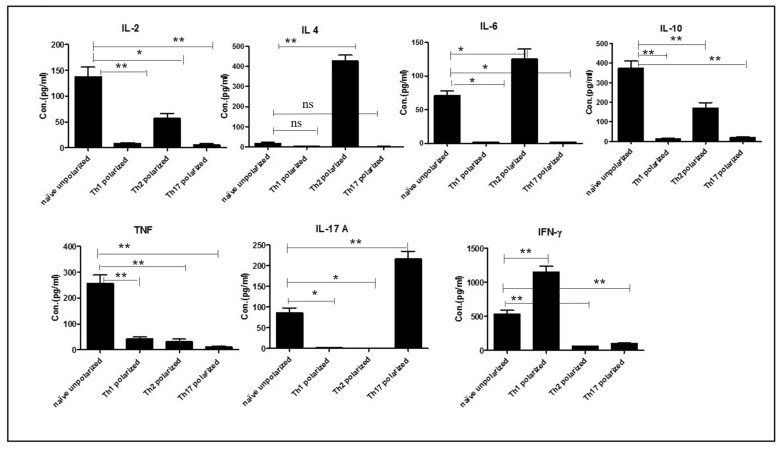
Naïve OT-II T cells were extracted from spleen and different lymph nodes of OT-II mice and these cells were cultured to polarize them, using OVA (20 μg/mL) and differentiating cytokines and antibodies to cytokines. After washing, differentiated cells were stimulated with IL-7 for a further two days to produce memory cells. Finally, cells were stimulated with OVA (10 μg/mL) for 72 h and further analysed using a cytokine bead array and flow cytometry to measure the concentration of cytokines secreted in culture supernatants. Single * (*p* < 0.05) and double ** (*p* < 0.01) in the graph represents the value statistical association among the dependent and independent variables and “ns” represents no statistical significance. The experiment in this study were performed in triplicates and the statistical association between different dependent and independent variables was analysed using *t*-test.

Naïve OT-II T cells were extracted from spleen and lymph nodes of OT-II mice and, over a 4-day period, differentiated into Th1, Th2, and Th17 in vitro in the presence of OVA protein and specific polarizing cytokines and anti-cytokine antibodies, which enables the naïve cells to differentiate into specific lineages. Subsequently, cells were supplied with IL-7 to further differentiate them into memory cells. As shown in Figure 1, following this treatment, Th1 polarized cells produced typical Th1 cytokines such as IFN-γ and marginal levels of TNF. In contrast, Th2 polarized cells produced a significant amount of Th2 cytokines, in particular IL-2, IL-4, IL-6, and IL-10, but no IFN-γ or IL-17A. In addition, Th17 polarized cells produced typical Th17 cytokines mainly IL-17A, but only marginal levels of the other cytokines (IFN-γ, TNF, IL-2 and IL-10). Naïve or undifferentiated cells produced IL-2, IL-6, IL-10, IL-17, IFN-γ, and TNF; but not IL-4 (Figure 1).

### 3.2. In Vivo Proliferative Response of Th-Polarised Cells to OVA-Expressing Eggs Following Ex Vivo Stimulation with the Same Antigen

**Figure 2:**
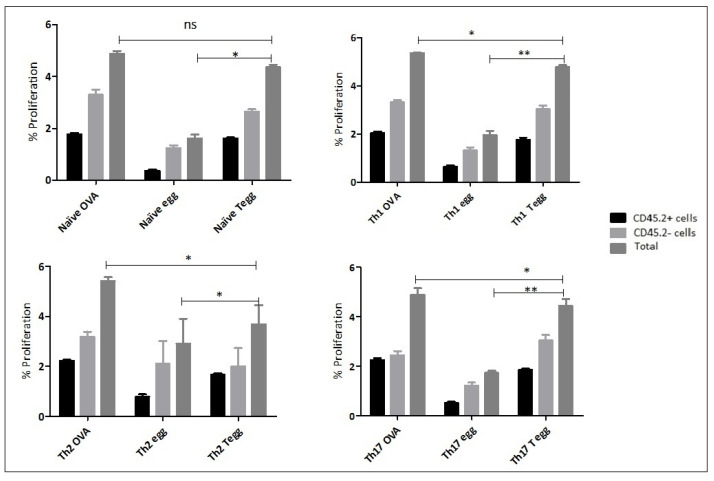
Naïve OT-II T cells were first differentiated into Th1, Th2 and Th17 cells using polarising cytokines and a total of 2 × 10^6^ each polarized cells were adoptively transferred into congenic mice. After 48 h, each recipient mice were challenged with either OVA protein (30 μg/mL) or 1000 OVA-expressing eggs or 1000 wild type eggs. After seven days, mice were culled to recover memory Th cells from spleen and lymph nodes. After recovery, the cells were re-stimulated in vitro with the same preparation as the initial in vivo stimulation and proliferation was assessed using the CFSE method and CD4 surface staining. The abbreviation naïve, Th1, Th2, and Th17 followed by words such as OVA, egg, and Tegg represents an initial injection of the cells into recipient mice and later challenged by the antigens, OVA protein, untransduced eggs, OVA expressing eggs, respectively. Single * (*p* < 0.05) and double ** (*p* < 0.01) in the graph represents the value statistical association among the dependent and independent variables and “ns” represents no statistical significance. The experiment in this study were performed in triplicates and the statistical association between different dependent and independent variables was analysed using *t*-test. The proportion (%) in the *Y*-axis represents the total number of proliferated T helper cells following antigen stimulation for each type of cells injected into the mouse and a challenge of that mouse with the different antigens (*X*-axis).

The aim of this experiment was to confirm that in vitro differentiated Th1, Th2 and Th17 memory T cells recovered from recipient mice following adoptive transfer, were able to proliferate after ex vivo re-stimulation. Following in vitro polarization of naïve OT-II T cells, into Th1, Th2, and Th17 phenotypes, each recipient mouse was injected I.V. with 2 × 10^6^ cells from each phenotype and the same number of naïve undifferentiated cells as a control and two days later, each group of mice was challenged either with OVA protein, OVA expressing eggs or wild type eggs through the same route of administration. Mice injected with Th1, Th2 and Th17 cells and followed by a challenge by OVA-expressing eggs showed a significantly higher rate of proliferation than mice injected with the same type of cell phenotypes but challenged with wild type eggs (Figure 2). Furthermore, naïve OT-II cells recovered from mice adoptively transferred with naïve OT-II cells could be induced to proliferate significantly more when restimulated with either OVA protein (positive control) or with OVA-transduced eggs, than with untransduced eggs. In the case of the Th2 polarised OT-II cells, the OVA protein induced significantly higher level of proliferation compared to the OVA-tranduced eggs. In the case of Th1 and Th17 cells background proliferative response when stimulating with non-transduced control eggs was very low, but in the case of the Th2 cells, this level of proliferation was close to the level induced by OVA-transduced eggs (Figure 2).

### 3.3. In Vivo Response of Th-Polarized Cells to OVA-Expressing Eggs

**Figure 3:**
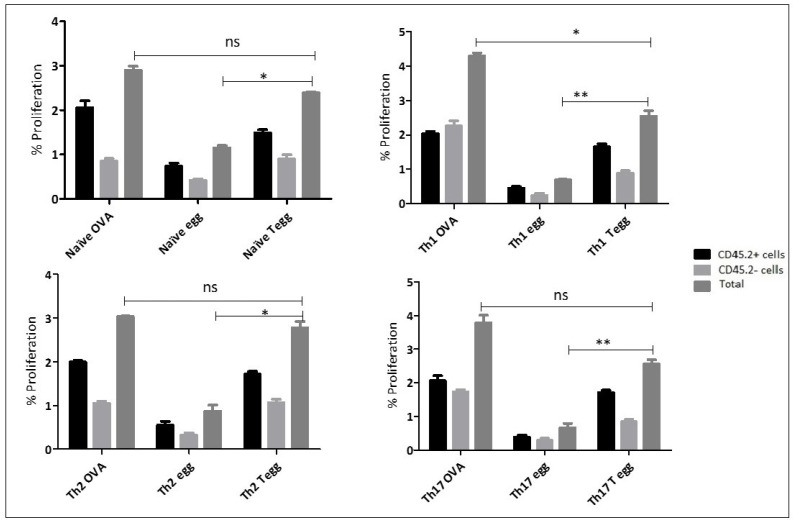
Adoptive transfer of Naïve, Th1, Th2, and Th17 cells and measuring proliferation rate in vivo. This figure depicts the proportion of naïve, Th1, Th2, Th17 cells following a challenge with either OVA protein at a concentration of 30 μg/mL or OVA-expressing eggs or wild type eggs at a concentration of 1000 eggs in both conditions. After a total of seven days, recipient mice were culled to recover memory cells from spleen and other lymph nodes and the proliferation of the cells was analysed directly based on the initial staining with CFSE and surface staining using specific CD4 markers after recovery and finally followed by a cytometry analysis to quantify the proliferation rate. The abbreviation naïve, Th1, Th2, and Th17 followed by words such as OVA, egg, and Tegg represents an initial injection of the cells into recipient mice and later challenged by the antigens, OVA protein, untransduced eggs, OVA expressing eggs, respectively. Single * (*p* < 0.05) and double ** (*p* < 0.01) in the graph represents the value of statistical association among the dependent and independent variables and “ns” represents no statistical significance. The experiment in this study were performed in triplicates and the statistical association between different dependent and independent variables was analysed using *t*-test. The proportion (%) in the *Y*-axis represents the total number of proliferated T helper cells following antigen stimulation for each type of cells injected into the mouse and a challenge of that mouse with the different antigens (*X*-axis).

Following in vitro polarization of naïve OT-II T cells, into Th1, Th2, and Th17 phenotypes, each recipient mouse was injected I.V. with 2 × 10^6^ cells from each phenotype and two days later, each group of mice was challenged with different antigens through the same route of administration. For this particular experiment, three antigens were used, namely: OVA protein, OVA-expressing eggs, and wild type eggs. In this study, two groups of T cells were characterized using the flow cytometric analysis based on their expression of CD4 and CD45.2. Indeed, in our system, OT-II cells can be differentiated from recipient T cells because they express CD45.2, while the T cells from the recipient mice express CD45.1. Mice injected with naïve OT-II cells followed by challenge with OVA-expressing eggs showed a significantly higher proliferation of cells compared to those challenged with wild type eggs (*p* < 0.05). Mice injected with naïve cells and followed by a challenge with OVA protein and OVA-expressing eggs showed no difference in the percentage proliferation (*p* > 0.05). In the second group, mice injected with Th1 cells and followed by challenge with OVA showed a significantly higher (*p* < 0.05) proliferation rate compared to those challenged with OVA-expressing eggs. Mice challenged with OVA-expressing eggs showed a significantly higher proliferation rate than those challenged with wild type eggs (*p* < 0.05). Mice injected with Th2 and Th17 and challenged with the OVA protein were similarly responding at a higher rate compared to mice challenged with wild type eggs. Similarly, as shown in Figure 3, Th2 and Th17 cells showed a higher response rate when challenged with OVA-expressing eggs than wild type eggs and the statistical association in both conditions shown a significance difference (*p* < 0.05). This revealed that polarized cells could proliferate to similar degrees following challenge in vivo when exposed to OVA (3–4%) and to transduced eggs (2–3%) (Figure 3).

### 3.4. Measuring Immune Memory Resilience to Pathogen Manipulation

**Figure 4:**
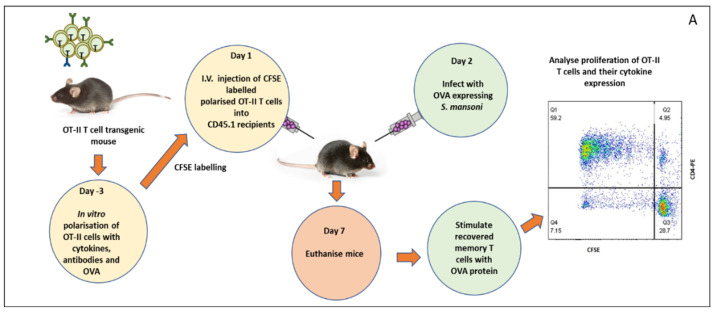

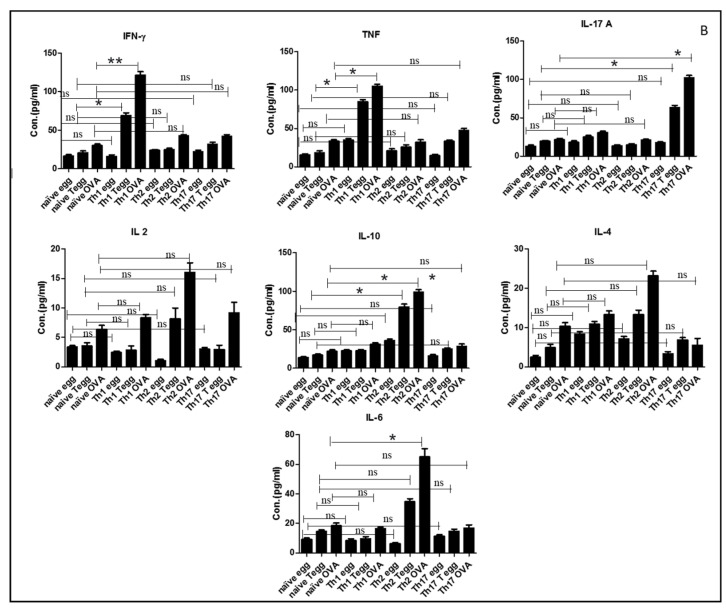
Th cytokines produced by Th-polarized memory T cells after infection with OVA expressing eggs and followed by ex vivo re-stimulation. After in vitro differentiation of naïve OT-II T cells into Th1, Th2, and Th17 cells, memory cells were further produced using IL-7 and a total of 2 × 10^6^ polarized memory T cells were transferred into each congenic mice (CD45.1, *n* = 6, I.V.). After two days, each congenic mouse was challenged with OVA-expressing eggs, OVA protein (30 μg OVA), and wild type eggs as a positive and negative control, respectively. After seven days, mice were culled to extract memory OT-II T cells and restimulated with OVA protein (20 μg OVA) (**A**) and finally, culture supernatants were analysed for detection of signature cytokines using cytometric bead array (**B**). Single * (*p* < 0.05) and double ** (*p* < 0.01) in the graph represents the value statistical association among the dependent and independent variables and “ns” represents no statistical significance. The experiment in this study were performed in triplicates and the statistical association between different dependent and independent variables was analysed using a *t*-test.

This experiment aimed at measuring the degree of immune memory resilience of Th polarized cells to pathogen manipulation and as shown in Figure 4A. Following injection of mice with naïve OT-II cells, Th1-polarized cells, Th2-polarized cells, or Th17-polarized cells, each group was challenged in vivo separately with OVA protein, OVA-expressing eggs, and wild type eggs and final recovered unpolarized (naïve) and Th memory cells were further cultured by stimulating with OVA protein. The Th memory cells were analysed by measuring their cytokine signatures. Not surprisingly, mice injected with naïve OT-II cells produce few cytokines whatever stimulus they were subjected to (Figure 4). In contrast, mice injected with Th1-polarised OT-II cells continue to produce their cytokine signatures such as IFN-γ and TNF at a significantly higher level compared to controls, following challenge with either OVA protein or OVA-expressing eggs. Similarly, mice injected with Th2-polaised OT-II cells continue to produce Th2 cytokines (IL-2, IL-4, IL-6, and IL-10) following a challenge with OVA and OVA-expressing eggs, in vitro following re-stimulation with OVA protein. Moreover, mice injected with Th17-polarised cells produced a significant amount of Th17 cytokines, in particular, IL-17A. The result of the cytokine analysis showed that all Th polarized, and memory cells produce their cytokine signatures following re-stimulation with OVA either as a protein or expressed in eggs. Th1, Th2, and Th17 polarized cells produced only Th1 cytokine, Th2 cytokine and Th17 cytokines respectively. Statistically, Th1 produced significantly more IFN-γ and TNF when exposed to OVA transduced eggs and similarly Th17 and Th2 produced significantly more IL-17 and IL-2 respectively when exposed to OVA transduced *S. mansoni* eggs (Figure 4).

## 4. Discussion

Following infection of a vaccinated animal with a pathogen possessing immunomodulatory properties, the quality of the immune memory induced by the vaccine and how it can be affected during a recall response is very important to the disease outcome. Indeed, parasites develop specific tactics to subvert the recall of an immune response, which influences the effectiveness of vaccination. The functional persistence and recall of memory CD4^+^ T cells allow the adjustment of the host’s immune response continuously, following exposure to different pathogenic infections [1]. To this end, understanding the development of immune memory resilience by Th polarised cells in the face of immune modulatory pathogens is very important for the development of effective vaccination strategies towards these immune modulatory pathogens.

In this study, we first demonstrated that OT-II cells could be fully differentiated into Th1, Th2 and Th17 cells since these cells produced their signature cytokines following in vitro treatment.

Previous finding suggest that a predominant Th1 response is induced in vitro following exposure to *S. mansoni* eggs. This result appears in contradiction with the observation that *S. mansoni* eggs induce a Th2 response during in vivo infection. One possible explanation for this discrepancy is that not all cells that were implicated in the induction of the expected Th2 response in vivo are present in vitro. For example, it is possible that some of the critical DC populations might not be present in sufficient numbers in the OT-II-based system. In addition, the OT-II cells can be stimulated not only by B cells but also by a range of other APCs present in cell cultures [12]. Another possibility is that OT-II cells are intrinsically biased towards Th1 responses. This has been suggested previously [13], at least when the OT-II cells are stimulated with B cells as APCs.

We subsequently demonstrated that these in vitro polarised memory Th cells can recognize OVA protein expressed by *S. mansoni* eggs and can respond by both proliferating and secreting specific cytokines such as IFN-γ, IL-17A, TNF, IL-10 and IL-6. Although we did not explicitly test whether OVA produced by the transduced parasites is glycosylated, we previously showed that it had an identical molecular weight to native OVA [12] and the fact that it is recognized by OT-II cells suggests that it is processed by APCs in a similar way to native OVA. We also showed that OVA protein expressed by *S. mansoni* parasite eggs is recognized by polarised T helper cells in vivo. Indeed, these cells proliferate significantly more in vivo when re-stimulated with OVA-transduced eggs compared to untransduced eggs. The only exception was Th2-polarised cells, which do appear to have a high background level of proliferation even when only stimulated with untransduced eggs and as a result, there is no significant difference with between the proliferation induced by transduced and untransduced eggs. The reason for this elevated background proliferation is currently unknown.

In the present study, under both in vivo and ex vivo stimulation conditions, memory T cells showed a significant proliferative response to OVA expressing *S. mansoni* eggs suggesting that they are able to not only survive but also continues to produce their cytokine signatures in the presence of the OVA antigen for extended periods of time. This is important, because CD4^+^ T cells require a prolonged exposure to the antigen for their differentiation into effector cells and memory development and survival [14,15,16]. Despite the long-term survival of memory cells in the presence of IL-7, there is a specific difference in the mechanism of their survival [17]. For example, engagement of MHC class II and T cell receptor signalling have implications in the functional capacities and long-term survival of memory CD4^+^ T cells [1]. The functional capacity of these memory CD4^+^ T cells is not irreversibly committed but it can be activated differentially during the recall immune response by changing the nature of the cytokine mediated signals present during the recall phase of the response [18]. Similarly, the pattern of gene expression by memory Th1 and Th2 cells have been found to develop functional flexibility. For example, Th1 and Th2 were cloned under the same or opposite polarising conditions and under the same polarising conditions, cells retained the capacity to produce originally imprinted cytokines, but when restimulated under opposite polarising conditions, cells produce alternative cytokines.

The capacity of T helper memory cells to retain the ability to produce their cytokine signatures was confirmed, and this study showed that immune memory resilience can indeed be measured in this way, and that the in vitro generated polarised Th cells remain unaffected by the parasite eggs. This despite the fact that *S. mansoni* eggs are well known for their ability to modulate the immune response towards a Th2 response following infection. The present finding is consistent with previous studies which showed that Th polarised cells can develop some degree of resilience to manipulation [19,20]. However, such studies were performed under in vitro conditions and it can therefore not be excluded that other in vivo controlling factors such as secretion of effector cytokines which have a regulatory effect in relation to the differentiation of CD4^+^ T memory cells under in vivo conditions. More importantly, in the case of such parasites which causes complex immune responses, an in vivo study is more important and valid than in vitro studies because the presence of DCs which plays a significant role in the initiation and polarisation of T cells responses and can be affected by the presence of *S. mansoni* eggs [21]. These DCs and the microenvironment they are in, might not be present under in vitro conditions.

The cytokine environment at the time of primary stimulation can alter the outcome of memory CD4^+^ T cell responses such as either promoting Th1 memory CD4^+^ T cells with proinflammatory responses or Th2 type memory CD4^+^ T cell responses [20,22]. Until the discovery of two other CD4^+^ T cell lineages, Th17, and Treg cells, the differentiation of naïve CD4^+^ T cells into specific lineages with specific effector function was considered an irreversible endpoints [23]. In contrast to the Th1 and Th2 phenotypes the Th17 cells are less stable and this leads to a higher degree of flexibility during their differentiation and this phenomenon has its own biological implication [23]. Indeed, the flexibility of these cells in terms of commitment to a particular phenotype might lead to lack of immune memory resilience in the face of an immunomodulatory pathogen. For example, it was reported that there is a cross-regulation between Th1, Th2, and Th17 during *S. mansoni* infection, with Th1 cytokines (such as IFN-γ), and Th2 cytokines (such as IL-4) inhibiting the production of Th17 cytokines (such as IL-17) [24].

It is well-established that following the acute phase of infection or during the first six weeks of infection, the immune response to *S. mansoni* is predominantly Th1, characterised by the abundant production of TNF-α, and IFN-γ [25]. However, soon after these 6 weeks, the parasite starts laying eggs, and the disease progresses into different organs mainly the liver and caused granulomas due to the immune response induced by the deposited *S. mansoni* eggs. At this stage Th2 immune response to egg antigens predominates and T cells produce high level of IL-4, IL-5, IL-13, and IL-10 [26]. The host-parasite interaction is very complex as they can involve both immunomodulatory mechanisms from the parasite and countermeasures from the host to resist these immunomodulatory effects. Following differentiation of naïve T cells in vitro into Th1, Th2, and Th17 phenotypes, we tested the degree of plasticity and resilience in the face of a parasite challenge after the polarised cells were transferred into recipient mice for in vivo study. The results showed that, Th1, Th2, and Th17 memory cells continued to produce their signature cytokines, when challenged by the parasite, suggesting that the parasite eggs were unable to manipulate the immune response of the differentiated T cells. At first sight this seems to contradict the well-established observations that the initial Th1 response against the parasite during the acute phase morphs into a Th2 response during the egg stage of the parasite. However, there are several major differences between our experiments and these observations, which may explain this apparent discrepancy.

First, we use fully differentiated memory cells and we do not know for sure in how far these can be compared to the memory cells present following the acute phase of the parasite or more importantly the vaccination using an effective adjuvant. Indeed, it is possible that the memory cells likely to be produced during the acute phase might not be as differentiated as these that we produce artificially in vitro with a cocktail of cytokines and antibodies to cytokines. If the cells generated following the acute phase of the parasitemia are not as differentiated, they may be less resilient to manipulation by the parasite, and hence could be manipulated during the egg-production phase. Secondly, unlike in our case, the antigen specificity of the Th2 immune response induced during the egg-phase could potentially be different to the antigen specificity of the immune response against the acute phase. Hence, the cells that are Th2 during the egg-phase of the infection could (at least theoretically) be different from these of the acute phase. In that case, there would be no need for the acute-phase memory cells to change their phenotype, they would just be superseded by the cells of the egg-phase. Of course, in our experiments this is not an option as all cells are specific for OVA and therefore, we are only considering a change in phenotype during the manipulation. This is a much greater ask of the parasite’s ability to manipulate the immune response. However, our experiments are still pertinent for vaccine development as they demonstrate that it might be possible to establish an immune memory response against an antigen that is resilient to manipulation and thus it is worthwhile to pursue this goal identifying both suitable antigens and effective adjuvants that promote full the differentiation of memory Th cells. Such vaccines would be able to withstand manipulation by the *S. mansoni* and hence protect the vaccinated host. Whether this can also be achieved for other pathogens remains to be explored.

## 5. Conclusions

In conclusion, we demonstrated for the first time, that in the face of the immune manipulative pathogen the immune response to a neutral model antigen such as OVA expressed by *S. mansoni* can’t modulate a fully mature immune memory Th response to this antigen, under the in vivo condition.

## Data Availability

The datasets used and/or analyzed during the current study available from the corresponding author on reasonable request.
